# Contextualizing and assessing the social capital of seniors in congregate housing residences: study design and methods

**DOI:** 10.1186/1471-2458-5-38

**Published:** 2005-04-18

**Authors:** Spencer Moore, Alan Shiell, Valerie Haines, Therese Riley, Carrie Collier

**Affiliations:** 1 Centre for Health & Policy Studies, Dept. of Community Health Sciences, University of Calgary, 3330 Hospital Dr. NW, Calgary, AB T2N 4T1, Canada; 2 Department of Sociology, 2500 University Dr. NW, University of Calgary, Calgary, AB T2N 1N4, Canada; 3 VicHealth Centre for the Promotion of Mental Health and Social Wellbeing, School of Population Health, University of Melbourne, level 5, 207 Bouverie St., Carlton, 3052, Australia; 4 Calgary Health Region, Healthy Living, SE Community Portfolio, Mental Health, NE Community Portfolio, Calgary, AB Canada

## Abstract

**Background:**

This article discusses the study design and methods used to contextualize and assess the social capital of seniors living in congregate housing residences in Calgary, Alberta. The project is being funded as a pilot project under the Institute of Aging, Canadian Institutes for Health Research.

**Design/Methods:**

Working with seniors living in 5 congregate housing residencies in Calgary, the project uses a mixed method approach to develop grounded measures of the social capital of seniors. The project integrates both qualitative and quantitative methods in a 3-phase research design: 1) qualitative, 2) quantitative, and 3) qualitative. Phase 1 uses gender-specific focus groups; phase 2 involves the administration of individual surveys that include a social network module; and phase 3 uses anamolous-case interviews. Not only does the study design allow us to develop grounded measures of social capital but it also permits us to test how well the three methods work separately, and how well they fit together to achieve project goals.

This article describes the selection of the study population, the multiple methods used in the research and a brief discussion of our conceptualization and measurement of social capital.

## Background

Research in gerontology has long demonstrated the importance of social relationships and support for the health and well-being of seniors [1-4]. More recent research in the health sciences has demonstrated the effects of place or spatial context on individual health [5,6]. The current project unites the ideas of the social-network and spatial contexts to examine the effects of both on the health of seniors living in residential housing arrangements. The idea of the social-network context proposes that seniors are embedded in a structure of inter-personal ties and that this structure can influence their behavior and access to valued resources; the idea of the spatial context suggests that seniors reside in certain locations and have certain opportunity structures available to them as a result of this location. The effects of the two contexts on health may not be not mutually exclusive. Our long-term research program, which the pilot initiates, aims to examine how residential environments and social networks may act independently to influence the health and aging experiences of seniors and how they may act on one another to influence opportunity structures in residential environments and the shape of seniors' social networks.

Using a mixed method approach moving along a qualitative – quantitative – qualitative sequence, the pilot project initiates our research program in the social environments of aging. As a pilot project on social capital and seniors, the research will be concerned first of all with feasibility issues related to working with this population of seniors: 1) Are seniors resident in congregate housing capable and willing to participate in the different phases of the study? 2) How acceptable and feasible is it to obtain data on all aspects of a senior's networks both inside and outside congregate housing residences? (3) What will the participation and response rates be for follow-up anomalous case interviews? In addition to piloting our work with seniors in Calgary housing residencies, the project's mixed method approach will allow us to (1) develop contextualized understandings of social capital that are tailored to seniors in congregate housing, (2) measure empirically the social capital among seniors living in different seniors' housing facilities, and (3) examine how our explanatory models can be improved through follow-up anomalous case studies.

While there is a considerable gerontological literature on community-dwelling seniors, little research has been devoted to the social ties of the institutionalized elderly whose experiences can differ considerably from those of community-based seniors [7]. By focusing research on the social capital and aging experiences of seniors resident in non-profit and publicly subsidized housing, the pilot research targets a unique population of seniors who could be considered liminal to these two other populations. Such seniors are no longer community-dwelling *per se *but they are still expected to be independent with only minimal support; they thus access resources and maintain social ties both inside and outside their residence.

### Why social capital?

Broadly speaking, there are two approaches to the concept of social capital: the communitarian and network. Perhaps, most evident in the work of the political scientist, Robert Putnam, and the public health research of Ichiro Kawachi, the communitarian approach to social capital emphasizes the shared values and norms of a group or a community [5,8,9]. As defined by Putnam, social capital refers to "the features of social organization, such as networks, norms, and trust that facilitate coordination and cooperation for mutual benefit [10]." Common to the communitarian approach is a vision of social capital as a common collective asset or public good equally available to all members of a group or community. In practice, the measurement of social capital becomes equated with levels of trust, reciprocity, or civic participation [8].

The network approach, on the other hand, views social capital as consisting of the resources to which individuals or groups have access through their own social networks: "social capital is the sum of resources, actual or virtual, that accrue to an individual or group by virtue of possessing a durable network of more or less institutionalized relationships of mutual acquaintance and recognition [11]." As evidenced primarily in the work of anthropologists and sociologists, the network approach to social capital emphasizes to varying degrees two elements: (1) the structure of an individual's or a group's network and (2) the resources to which individuals or groups have potential access given the structure and composition of those networks [8,12-15].

Despite the promise that social capital holds for health promotion, interventions seeking to improve social capital have been slow to develop. One possible reason for this is that social capital's operationalization and measurement in the health sciences has been inconsistent and controversial [16]. The measurement of social capital in the health sciences has commonly relied on readily available secondary data on trust, reciprocity, or civic participation. Individual-level responses are aggregated to the neighborhood, community, state, or national level to determine that unit's level of social capital. What appears lacking in the health sciences research are (1) measures of social capital that are tailored to the populations which they address and (2) measures of social capital that derive from network analysis methods. The project, although concerned with feasibility issues, is designed to improve overall research in the area of social capital and health by addressing these two weaknesses.

## Methods/Design

### Sample

The pilot study will be conducted in five of sixty-nine public and non-profit congregate housing residences in Calgary chosen in partnership with the Calgary Health Region. Inclusion criteria for sites will involve (1) the residence having a seniors resource nurse (SRN) who visits regularly and (2) the SRN having a strong working relationship with the residents and management of the residences. Based on these criteria along with recommendations from SRNs, residence managers will be contacted and asked if they would agree to participate. Since it is a one-year pilot project, the number of sites is limited to five.

### Phase 1: focus groups

The first phase of the research will consist of gender-specific focus groups. Two gender-specific focus groups will take place in each facility. The decision to stratify according to gender is based on findings suggesting the differential distribution of social capital between genders [17,18]. In addition, preliminary field research has shown a majority of women at these locations. A stratified sample will allow us to ensure an adequate number of men.

The sampling frame for focus group participants will be the complete list of suite numbers of each facility. To generate a stratified random sample, suite numbers for each facility will be divided according to male or female resident to generate a stratified random sample. For suites containing couples, each resident will be listed separately. Random samples help reduce the biases that can result from using sign-up sheets and non-random methods [19]. Based on the list of randomly selected men and women, we will invite individuals from the top of the list until 15 – 20 residents for each group have agreed to participate or until we have exhausted the list.

The focus group script will consist of questions falling into three broad categories: 1) the meaning of social networks and social relations, 2) the identification and importance of social resources, and 3) perceptions of intra- and extra-facility trust and reciprocity. Focus group discussion will last for about 20 minutes in each category with the overall focus group lasting approximately 60 to 90 minutes. Discussions will be taped and transcribed in full.

Using content analysis, the focus group transcripts will first of all be examined to identify the key resources, individuals, and community-related events that seniors in congregate housing residences find important to them. These findings will be integrated into the resource generator module of the individual questionnaires. In addition, semantic and thematic analyses of the focus group transcripts will be undertaken to understand the meaning and importance of various types of social ties present in the residences, the levels of social support for seniors available, levels of community involvement, and other topics and themes that emerge from focus group discussions.

### Phase 2: individual questionnaires

During the second phase of data collection, all seniors residing in the five facilities will be invited to complete a face-to-face interview. The interview will consist of six components containing questions about respondents': (1) sociodemographic characteristics (e.g. income and education), (2) sense of mastery, (3) move into the congregate housing residence, (4) social capital as measured from a communitarian approach, (5) social network and the resources that flow through this network, and (6) health status and health service utilization. The health status component will consist primarily of the Short Form 36 (SF-36) [20].

The two components of the survey on social capital – the communitarian and network modules – will allow us to explore the potential advantages and disadvantages of each approach for understanding the social capital of seniors and its effects on their health and well-being. The communitarian-approach module will generate data on trust, reciprocity, and civic participation. The General Social Survey (GSS) question used by Kawachi et al. "Do you think most people would take advantage of you if they got a chance?" [5] will be used to assess perceptions of trust. Following Kawachi, reciprocity will be assessed using the percentage who agree with the statement that "most people try to be helpful." The questions on trust and reciprocity will be supplemented by adding the phrase "in this residential home" to situate the measures of trust and reciprocity in that particular residential environment. Civic participation will be assessed by asking respondents about the types of associations to which they belong and their degree of participation in those associations. This question will be supplemented by an additional one asking respondents about their participation in associations or events in their residential facility. Following the coding and analysis of the focus group sessions, additional questions based on that analysis will be added to this component of the survey.

The network module of the questionnaire will consist of two instruments: 1) name generator/name interpreter sequences and 2) a resource generator. Name generators are questions that ask respondents to identify members of their social networks (alters). Different name generators tap different sectors of respondents' networks. To tap network members inside and outside the housing residence, we will use 4 name-generator questions: (1) who have you discussed important matters with in the last 6 months?, (2) who have you socialized with in the last 6 months?, (3) who do you feel attached to?, and (4) who do you know well enough to call up and talk with on the phone but who you don't know *really *well? Name interpreter questions are follow-up questions that generate information about 1) the characteristics of the respondent's alters (e.g., age, gender, marital status), 2) the nature of the respondent-alter relationship (e.g., role relationship, how often they see each other, how long they have known each other) and 3) the nature of the alter-alter relationships (e.g., do alters know each other).

Resource generators do not elicit the names of network members. Instead they ask respondents about access to specified resources through individuals whom they know in some capacity [21,22]. The resources that will be included in the resource generator will be identified through the phase one qualitative research and analysis. Resources that seniors may find important include "access to a car" or "knowing someone who can get them to a grocery store." Follow-up questions will ask whether the resources are accessed through family members, friends, or acquaintances. Resource generators have only recently been posited as an accurate instrument for assessing the social capital of individuals [21,22] and have yet to be tailored to senior populations.

### Phase 3: anomalous-case interviews

During the third phase of data collection, a listing of anomalous cases will be created for each facility. We will use regression analysis to identify statistical outliers in the regression models used to test our hypotheses about social capital and its effects on the health of seniors. Statistical outliers might be identified on the following bases: their residual values, deviance from their predicted values, and influence over the regression models themselves [23]. For example, anomalous cases may be identified on the basis of their statistical deviance from the hypothesized social capital – health association. If certain individuals with low social capital maintain high reported health, we would like to know why. Were survey items valid? Were they reliable? Or, are there important features of social network and residential contexts that are currently being missed in the literature on social capital and health.

Once the list of individuals who represent anomalous cases has been created for each facility, a random sample of individuals will be taken from this list. Semi-structured interviews will be conducted with those selected and who agree to participate. While the specific questions to be included will only be developed after the analysis of phase one and phase two data, interview questions might center around participation in intra- or extra-residential events or ties to individuals who did not appear in the network questions, or their access to resources in Calgary. Follow-up interviews with anomalous cases in these areas will allow us to build theory, improve measures, and test our measurement instruments [23].

## Competing interests

The author(s) declare that they have no competing interests.

## Authors' contributions

All authors participated in the design of the study. SM coordinated the study and drafted the manuscript. All authors read drafts of the manuscript, made comments and suggestions, and approved the final version.

## Pre-publication history

The pre-publication history for this paper can be accessed here:


